# Biocontrol Potential of *Trichoderma* spp. Against *Phytophthora ramorum*

**DOI:** 10.3390/pathogens14020136

**Published:** 2025-02-02

**Authors:** Elisa Becker, Nirusan Rajakulendran, Simon Francis Shamoun

**Affiliations:** 1 Pacific Forestry Centre, Canadian Forest Service, Natural Resources Canada, 506 West Burnside Road, Victoria, BC V8Z 1M5, Canada; simon.shamoun@nrcan-rncan.gc.ca; 2 Independent Researcher, Toronto, ON M3H, Canada; nirusan.rajakulendran@gmail.com

**Keywords:** *Phytophthora ramorum*, sudden oak death, biocontrol, biological control agents, *Trichoderma*, mechanisms of action, antagonistic metabolites, in vitro assay, dual culture assay

## Abstract

*Phytophthora ramorum*, the cause of Sudden Oak Death and related diseases, threatens over 130 tree and shrub species. We evaluated the biocontrol potential of isolates from nine *Trichoderma* species against *P. ramorum* using growth-rate studies, dual-culture assays, and culture-filtrate assays. Results showed significant variation in *Trichoderma* growth rates and biocontrol potential. Some isolates exhibited rapid growth, effective overgrowth, and lethal effects against *P. ramorum* and produced potent antagonistic metabolites. Faster growth rates only partially correlated with biocontrol efficacy, indicating that factors beyond growth, such as metabolite production, play significant roles. Notably, isolates of *T. koningii*, *T. viride*, and the commercial product SoilGard™ (*T. virens*) showed promising efficacy. We calculated a combined biocontrol variable to rank isolates based on vigour and efficacy to aid in identifying promising candidates. Our findings support the use of *Trichoderma* spp. as biocontrol agents against *P. ramorum* and underscore the need for a multifaceted approach to selecting and optimizing isolates. Our evaluation demonstrated the importance of using different assays to assess specific mechanisms of action of biocontrol candidates. Future research should further explore these interactions to enhance the sustainable management of *P. ramorum*.

## 1. Introduction

*Phytophthora ramorum*, Werres, de Cock & Man in’t Veld [1], a globally significant invasive plant pathogen, causes substantial ecological and economic damage through diseases such as Sudden Oak Death in the U.S. and Sudden Larch Death and ramorum blight in Europe [2]. Its broad host range threatens forest biodiversity and the viability of the nursery industry. This oomycete affects over 130 species of trees and shrubs, including important nursery crops such as rhododendron, viburnum, and lilac, as well as forest species like maple and oak [3,4]. The pathogen produces sporangia on infected leaves and twigs, releasing motile zoospores that encyst and penetrate hosts under suitable conditions, initiating new infections [1]. In addition to causing foliar and stem diseases, *P. ramorum* survives in soil and can infect rhododendron roots, demonstrating its broad pathogenic capabilities [5,6].

The long-distance spread of *P. ramorum* has predominantly occurred through the transport of infected nursery stock. It has been isolated from diseased plants in nurseries across California, Oregon, Washington, and British Columbia (B.C.), with infected plants shipped to 32 U.S. states between 2003 and 2007 [3]. It has established in Florida and Mississippi, as evidenced by repeated detections in baiting surveys. In Europe, *P. ramorum* is considered an introduced pathogen found in nurseries and woodland gardens. This has led to quarantine regulations aimed at reducing further spread in North America and Europe. Despite detection in hundreds of nurseries and several wildland areas, its spread in natural environments in western North America remains limited to areas of California and southwestern Oregon. In B.C., *P. ramorum* is a quarantined pest posing a moderate risk to native forest ecosystems [7]. Since its detection in several B.C. nurseries in 2003, eradication actions have been mandated at all sites with infected plants [4,8]. While there are no cures for infected plants, several *P. ramorum* control measures are registered in the U.S. and Canada for preventive use on susceptible plants. These measures, alongside quarantine policies, focus on reducing inoculum levels and limiting human-facilitated pathogen spread. Minimizing the risk of infection is the primary defence against the spread of the pathogen to uninfected plants [9].

*Trichoderma* spp. are fast-growing, opportunistic fungi that function as avirulent plant symbionts. Their antagonistic abilities against plant pathogens have promoted their use as biological control agents [10,11,12,13]. They have shown potential as biocontrol agents against certain *Phytophthora* species [14]. For example, apple seedlings co-cultured with *Phytophthora cactorum* and *Trichoderma* spp. showed significant reductions in root damage and increased plant weight compared to those infected with *P. cactorum* alone [15].

Mycoparasitism, a process where one fungus parasitizes another, often involves the secretion of cell wall-degrading enzymes that hydrolyze the cell walls of target fungi or oomycetes [11]. Antibiosis, the production of inhibitory substances, is another major biocontrol mechanism employed by *Trichoderma*, which produces various antimicrobial compounds [16]. Other modes of action of *Trichoderma* strains include promoting plant growth and plant defence mechanisms and competing for nutrients and space [16,17,18,19]. To address the need for effective biocontrol agents against *P. ramorum*, this study investigates the potential of *Trichoderma* spp.

The objective of this study was to investigate the antagonistic properties of isolates from nine *Trichoderma* species, including three commercial biocontrol products, against *P. ramorum*. We conducted three in vitro biological assays to screen *Trichoderma* isolates as potential biological control candidates against *P. ramorum*.

- **Analysis of Temperature-Dependent Growth Rate**: We began by performing a study of temperature-dependent growth rate on 51 *Trichoderma* isolates, hypothesizing that the growth rate of *Trichoderma* correlates with its biocontrol efficacy against *P. ramorum*. *Trichoderma* isolates that grow and colonize substrates rapidly could compete more effectively against *P. ramorum.*
- **Dual-Culture Assay for Direct Interaction**: Next, we assessed the direct interaction, or mycoparasitism, of *Trichoderma* with *P. ramorum*. This assay used a dual-culture approach to measure the rates at which *Trichoderma* overgrew and killed an existing *P. ramorum* culture through direct interaction.
- **Antibiosis Microplate Assay for Antagonistic Effects**: Additionally, we investigated the antibiosis of different *Trichoderma* isolates with respect to *P. ramorum* by testing sterile culture filtrates of *Trichoderma* isolates in a novel in vitro microplate assay to assess their antagonistic effects on *P. ramorum* germination and growth.

To conclude, we calculated a combined biocontrol variable to represent the general vigour and overall efficacy of the *Trichoderma* isolates.

## 2. Materials and Methods

### 2.1. Analysis of Temperature-Dependent Growth Rate

To measure the temperature-dependent linear growth rates of *Trichoderma* isolates (Table 1), a growth-rate study was performed at two temperatures: 10 °C and 20 °C. Agar plugs were taken from the leading edges of fresh cultures grown on potato dextrose agar (PDA) plates at 20 °C for 2 days. Growth-rate cultures were initiated on Petri plates (100 mm × 15 mm) containing 25 mL of PDA by placing an agar plug 5 mm from the edge of the plate. The plates were incubated at room temperature (~20 °C) for 4–8 h to allow the fungi to grow from the plug onto the agar surface. The plates were then transferred to incubators set at either 10 °C or 20 °C in darkness. Measurements of mycelial growth were taken along predetermined axes at 24, 48, 72, and 96 h after the acclimation period. Growth during the acclimation period was not included in the calculation of the overall growth rate. The linear growth rate was expressed in mm/day.

### 2.2. Dual Culture Assay for Direct Interaction

**Figure 1:**
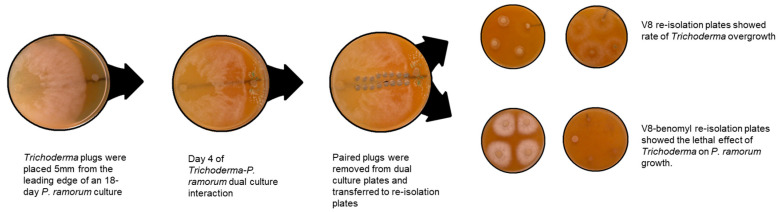
*Trichoderma* isolates in dual culture with 18-day-old *P. ramorum* cultures.

A dual-culture assay was used to evaluate direct interactions between the antagonist and pathogen, following the method of Goldfarb et al. [20]. To allow *P. ramorum* (NA1 lineage, accession 5073, isolated from *Rhododendron*) to establish before introduction of the faster-growing *Trichoderma* cultures, placements were timed accordingly (Figure 1). Each plate containing 15% V8 agar amended with 0.15% CaCO_3_ was bisected by a line drawn on the plate. An agar plug with an active culture of *P. ramorum* was placed on the center line, approximately 5 mm from the edge of the plate. The plate was incubated at 20 °C for 18 days or until the *P. ramorum* culture reached a diameter of 5.5 cm. To mark the farthest extent of *P. ramorum* growth, a line was drawn perpendicular to the center line. An agar plug of *Trichoderma* from the leading edge of a fresh 2-day-old PDA culture was placed on the center line, with its closest edge 5 mm from the marked extent of *P. ramorum* growth. After 4 days of dual culture growth, eight pairs of 6 mm plugs (one from each side of the center line), spanning a distance of 48 mm within the *P. ramorum* growth area, were collected. One plug from each pair was transferred to a plate containing 15% V8 agar amended with 0.15% CaCO_3_ to test for the presence of viable *Trichoderma*. The corresponding plug was transferred to a plate containing 15% V8 agar amended with 0.15% CaCO₃ plus 100 ppm benomyl (to inhibit *Trichoderma* growth) to test for the presence of viable *P. ramorum*. Re-isolation plates were monitored for the presence of *P. ramorum* or *Trichoderma* for up to 10 days (Figure 1).

The two parameters measured in this assay were the rate of overgrowth of the pathogen by the candidate *Trichoderma* culture and the rate of lethal effect. The rate of overgrowth was determined by measuring the linear distance on the dual culture plates over which *Trichoderma* overgrew the established *P. ramorum* culture. Growth of both *P. ramorum* and *Trichoderma* on their respective re-isolation plates indicated that *Trichoderma* had overgrown *P. ramorum* but had not killed it. The rate of lethal effect measured how effectively candidate cultures of *Trichoderma* spp. could kill *P. ramorum*. The absence of *P. ramorum* growth on the benomyl-amended re-isolation plates indicated it had been killed by the *Trichoderma* isolate (Figure 1).

### 2.3. Antibiosis Microplate Assay for Antagonistic Effect

#### 2.3.1. Phytophthora Ramorum Sporangia Production

For sporangia production, fresh agar plugs of *P. ramorum* were transferred into 60 mm plates containing clarified 15% V8 broth amended with 1% CaCO_3_ and incubated for 3–5 days. After incubation, these liquid cultures were washed with sterile distilled water (sdH_2_O) to remove nutrient media, resuspended in sdH_2_O, and incubated for 3 days to induce sporangia production. To separate sporangia from mycelium, cultures were filtered through three layers of sterile cheesecloth. To stimulate zoospore release, the sporangia suspension was chilled at 4 °C for 1 h, then incubated at room temperature for 1–2 h, with zoospore release confirmed by microscopy. An aliquot of the zoospore suspension was transferred into a centrifuge tube and vortexed for 30 s to induce encystment. Encysted zoospores were counted using a hemocytometer, and appropriate dilutions were prepared in clarified 15% V8 broth amended with 0.15% CaCO_3_.

#### 2.3.2. Production of Trichoderma Filtrates

*Trichoderma* isolates were cultured from agar plugs on PDA plates incubated at 20 °C in the dark for 2–3 days. Plates were subsequently placed under constant light until the cultures started to sporulate. Spore suspensions were prepared by flooding the plates with sdH_2_O and mixing with equal volumes of 20% glycerol, and the suspensions were then stored at –20 °C. On the day of inoculation, spores were counted using a hemocytometer and 100 mL of potato dextrose broth (PDB) was inoculated with *Trichoderma* spores to a final concentration of 10,000 spores/mL. Liquid cultures were incubated at room temperature with constant shaking at 130 rpm for 5 days. Mycelium was collected by filtering through three layers of cheesecloth and washed with 25 mL of sdH_2_O. Washed mycelium was aseptically transferred into 250 mL flasks containing 100 mL of sdH_2_O and incubated at room temperature with constant shaking at 130 rpm for 2 days. This culture filtrate was separated from mycelium by first pouring the liquid through three layers of cheesecloth, then filtering it through a 0.2 μm syringe filter to remove spores and mycelial fragments.

#### 2.3.3. Antibiosis Microplate Assay

**Figure 2:**
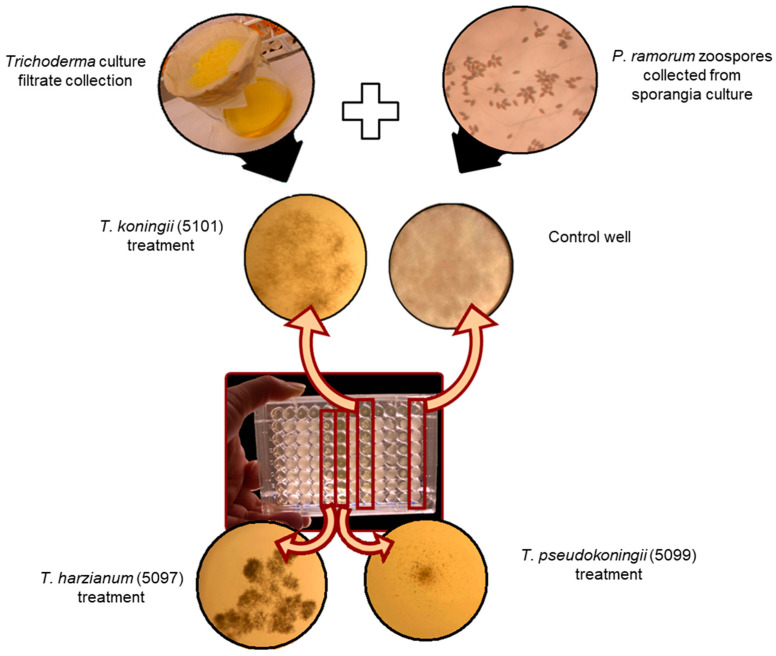
Microplate assay showing the effects of sterile *Trichoderma* culture extracts on *P. ramorum* germination and growth. The control wells contained only *P. ramorum* zoospores and showed uninhibited germination and growth, while the other wells also contained extracts of *Trichoderma* isolates and showed variable inhibition of *P. ramorum* growth relative to the controls. These examples show the least relative inhibition of *P. ramorum* in the top left (isolate 5101) and the most inhibition in the bottom right (isolate 5099).

To test the activity of sterile filtrates, 100 μL of *P. ramorum* zoospore suspension (1000 zoospores/mL in 15% clarified V8) was mixed with 100 μL of *Trichoderma* culture filtrate in 96-well microplates (Figure 2). To generate the positive controls, 100 μL of sdH_2_O was substituted for *Trichoderma* culture filtrate. To generate the negative controls, 100 μL of 15% V8 broth was substituted for the *P. ramorum* zoospore suspension and mixed with 100 μL of *Trichoderma* filtrates. Absorbance at 650 nm was measured at 0, 24, 48, and 72 h. The percent inhibition was calculated using the following formula: % inhibition = [(Control − Treated)/Control] × 100.

### 2.4. Statistical Analyses

Statistical analyses were performed using R and RStudio with packages including *tidyr, dplyr*, *psych*, *ggplot2*, and *RColorBrewer* [21,22,23,24,25,26,27]. To create a combined biocontrol variable, values of each relevant variable were standardized (mean of 0 and standard deviation of 1) using the ’scale’ function, then combined into a single composite score by averaging the standardized variables.

## 3. Results

### 3.1. Analysis of Temperature-Dependent Growth Rate

**Figure 3:**
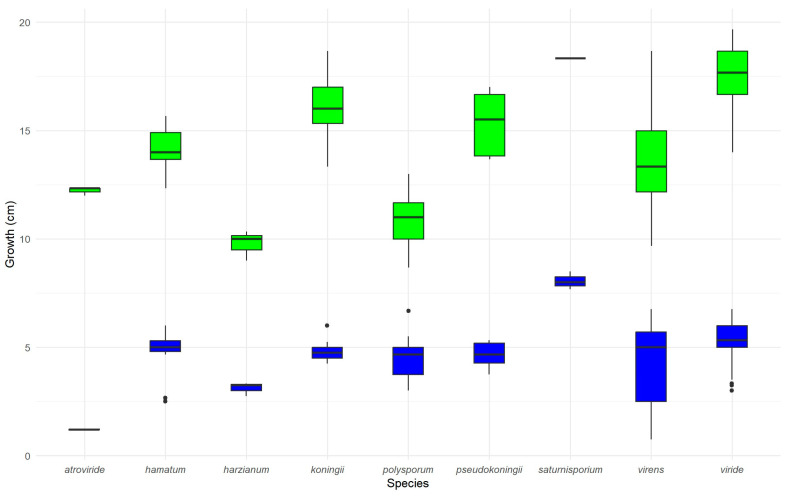
Box plots showing the average radial growth of *Trichoderma* isolates, grouped by species, on PDA at two temperatures (10 °C in blue and 20 °C in green) after 3 days. The numbers of isolates per species were as follows: *atroviride* (1), *hamatum* (6), *harzianum* (1), *koningii* (9), *polysporum* (11), *pseudokoningii* (2), *saturnisporium* (1), *virens* (5), and *viride* (15). Each isolate had 3 replicates. Boxes represent the interquartile range (IQR), with medians indicated by horizontal lines. Whiskers extend to 1.5 times the IQR, and outliers are individual points.

The mean radial growth of *Trichoderma* isolates at 10 °C after 3 days was 4.76 ± 1.25 cm (Figure 3). At 20 °C, the mean growth after 3 days was 14.72 ± 3.03 cm. The rate of linear growth ranged from 8.0 to 19.4 mm/day among 51 isolates (each with three replicates), with isolate 5035 (*T. viride*) growing the fastest.

### 3.2. Dual Culture Assay for Direct Interaction-Rate of Overgrowth

**Figure 4:**
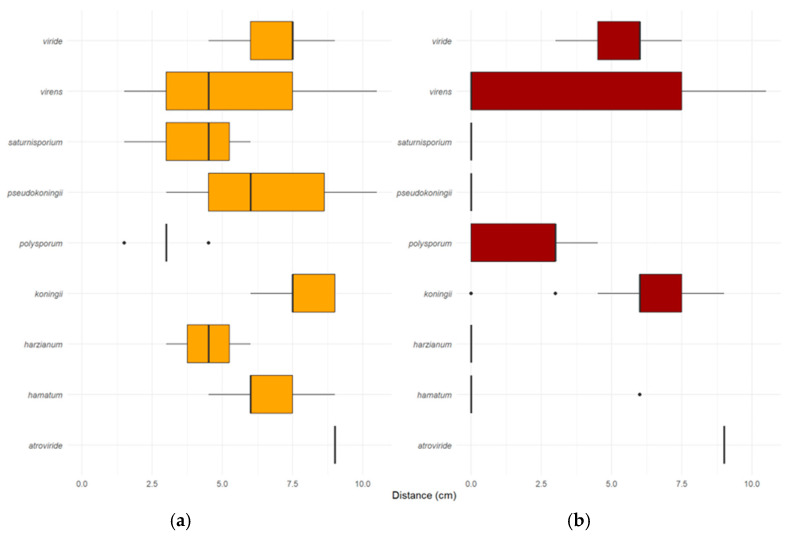
Box plots showing the average overgrowth and lethal effects of *Trichoderma* species in dual culture with 18-day-old *P. ramorum* cultures, as measured in cm after 3 days. Panel (**a**) shows the average radial growth of *Trichoderma* spp. on the surface of media with established *P. ramorum*. Panel (**b**) displays the average distance over which *Trichoderma* species killed *P. ramorum*. Boxes represent the interquartile range (IQR), with medians indicated by horizontal lines. Whiskers extend to 1.5 times the IQR, and outliers are individual points.

Overgrowth was assessed by the ability of *Trichoderma* cultures to overgrow an established *P. ramorum* culture (Figure 4). The mean overgrowth distance after 3 days was 5.95 ± 2.31 cm (Figure 4a). The commercial product SoilGard™ (*T. virens*) exhibited the highest overgrowth rate, at 10.5 mm/day.

### 3.3. Dual Culture Assay for Direct Interaction-Rate of Lethal Effect

The rate of lethal effect described the ability of *Trichoderma* isolates to mycoparasitize and kill *P. ramorum* through direct interactions (Figure 2). SoilGard (*T. virens*) demonstrated the highest rate of lethal effect at 10.5 mm/day, consistent with its high rate of overgrowth (Figure 4b). The ability of *Trichoderma* spp. to overgrow *P. ramorum* did not necessarily translate to a lethal effect. For example, *T. hamatum* had an average overgrowth rate of 6.0 mm/day; however, none of its isolates were able to mycoparasitize and kill *P. ramorum*. Similarly, *T. polysporum* (5003, 5018, 5025, 5026, 5034), *T. virens* (5095, 5096, 5098), *T. koningii* (5101), *T. pseudokoningii* (5093, 5094), and *T. harzianum* (5097) coexisted with *P. ramorum* without having a lethal effect on it. In addition, no *Trichoderma* isolate exhibited a lethal effect on *P. ramorum* from a distance. *Trichoderma* isolates had a lethal effect on *P. ramorum* only after overgrowing it.

### 3.4. Antibiosis Microplate Assay for Antagonistic Effect

**Figure 5:**
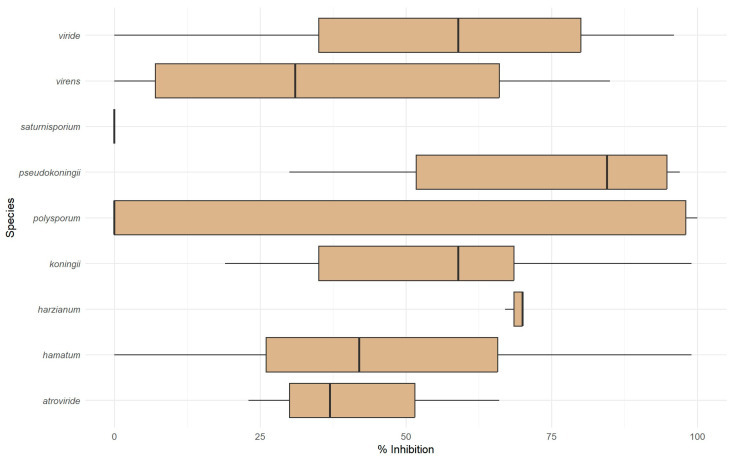
Box plot illustrating the inhibition by *Trichoderma* culture filtrates of *P. ramorum* zoospore germination and growth. The *x*-axis shows percent inhibition of treated *P. ramorum* compared to the control (0–100%). Boxes represent the interquartile range (IQR), with medians indicated by horizontal lines. Whiskers extend to 1.5 times the IQR, and outliers are displayed as individual points.

Culture filtrates collected under starvation conditions were tested for their inhibitory effects on *P. ramorum* (Figure 5). The average inhibition of *P. ramorum* growth by sterile culture filtrates of *Trichoderma* isolates was 49 ± 35%. The following isolates produced culture metabolites that inhibited *P. ramorum* growth by more than 75%: *T. virens* (SoilGard), *T. hamatum* (T382), *T. koningii* (5101), *T. pseudokoningii* (5099), *T. viride* (5008), and *T. polysporum* (5017, 5032, 5033, 5016, and 5001).

### 3.5. Correlation of Assay Variables

**Figure 6:**
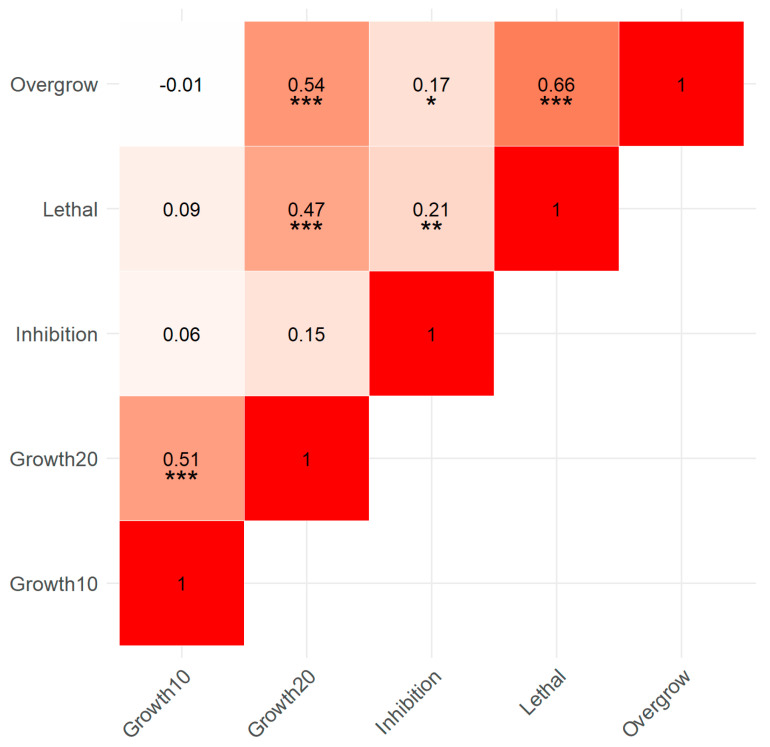
Correlation matrix showing Spearman correlation coefficients between all growth and biocontrol activity variables (growth of *Trichoderma* at 10 °C and 20 °C, overgrowth of *P. ramorum*, lethal effect on *P. ramorum*, and percent inhibition of *P. ramorum*). Darker shades indicate stronger correlations. Significance levels: *** (*p* < 0.001), ** (*p* < 0.01), * (*p* < 0.05).

A strong positive correlation was observed between the growth of *Trichoderma* isolates at 10 °C and their growth at 20 °C (Figure 6; r = 0.506, *p* < 0.001). Likewise, a strong positive correlation (r = 0.544, *p* < 0.001) suggested that increased growth of *Trichoderma* isolates at 20 °C was associated with increased overgrowth of *P. ramorum*. The strongest positive correlation (r = 0.657, *p* < 0.001) indicated a significant relationship between the overgrowth and the lethal effect on *P. ramorum* exerted by *Trichoderma*. A moderate positive correlation (r = 0.214, *p* = 0.008) suggested that higher values of percent inhibition were associated with higher lethal effect values. A moderate positive correlation (r = 0.167, *p* = 0.039) indicated a relationship between percent inhibition and overgrowth of *P. ramorum* by *Trichoderma*.

### 3.6. Combined Biocontrol Variable

**Figure 7:**
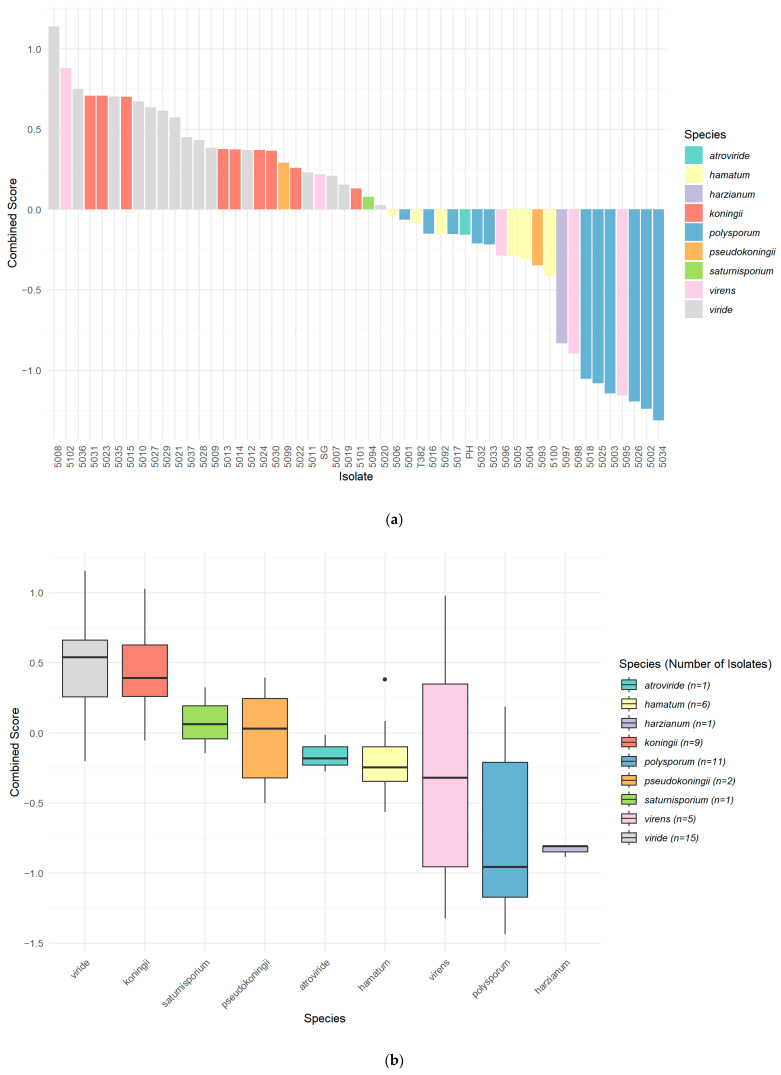
Mean combined scores of *Trichoderma*, as calculated from standardized variables related to biocontrol activity against *P. ramorum* (growth at 10 °C and 20 °C, overgrowth, lethal effect, and percent inhibition). In (**a**), scores of individual isolates are arranged from highest to lowest, with colors indicating species. Each bar represents the average score of an isolate based on three replicates. In (**b**), boxes show the distribution of scores for each species group, with the median as the midline, whiskers representing the interquartile range (IQR), and dots as outliers. Each isolate had 3 replicates. The number of isolates per species is shown in the legend.

A combined variable representing the relative general vigour and biocontrol efficacy of the isolates was developed. This was done by standardizing and scaling each of the relevant variables (growth of *Trichoderma* at 10 °C, and at 20 °C, overgrowth of *P. ramorum*, lethal effect on *P. ramorum*, and percent inhibition of *P. ramorum*) and then combining them into a single composite score. The combined biocontrol variable was used to rank each isolate (Figure 7a) and species group (Figure 7b).

## 4. Discussion

*Trichoderma* spp. act as potent antagonists against plant pathogens through various direct and indirect modes of action, including mycoparasitism, antibiosis, lytic enzyme secretion, and competition for space and nutrients [10]. Through direct mechanisms such as mycoparasitism, the antagonist secretes lytic enzymes, antibiotics, and other toxic metabolites that synergistically antagonize the pathogen. Ideal biocontrol agents exhibit most of these characteristics, enabling effective control of target pathogens [19,28,29].

Given these diverse biocontrol mechanisms, our study assessed the effectiveness of various *Trichoderma* isolates against *Phytophthora ramorum*, a destructive pathogen causing significant nursery and forestry diseases. We focused on the isolates’ growth rates and specific mechanisms of action to identify promising candidates for biocontrol applications against *P. ramorum*. Specifically, we evaluated 51 isolates from nine *Trichoderma* species using criteria reflecting various desired biocontrol abilities or traits.

In our investigation, we hypothesized that the growth rate of *Trichoderma* isolates would be directly correlated with their biocontrol efficacy against *P. ramorum*. Our *Trichoderma* growth rate study indicated a wide variation among the 51 isolates screened, with growth rates at 20 °C ranging from 8.0 to 19.4 mm/day. Notably, 5035 (*T. viride*), the fastest-growing isolate, showed significant biocontrol potential, suggesting that rapid colonization through competition for space is an effective mechanism of action against *P. ramorum*. The correlation between growth rate and biocontrol efficacy was inconsistent, as several fast-growing isolates did not exhibit comparable biocontrol efficacy. While competition for space and nutrients is a classical mechanism of biological control [30], our results suggest that multiple mechanisms, including antibiosis and mycoparasitism, in addition to growth rate, contribute to the biocontrol capabilities of *Trichoderma* against *P. ramorum*.

Mycoparasitism involves direct interactions with the pathogen, such as coiling around the pathogen’s hyphae, penetration, and secretion of lytic enzymes [31,32,33]. These enzymes break down the cell walls of the pathogen, leading to its disintegration [33]. In our study, *T. atroviride*, *T. koningii*, and *T. virens* demonstrated effective mycoparasitism by overgrowing and directly killing *P. ramorum* in dual culture (Figure 1 and Figure 4a,b). This aligns with the results of studies showing that production of lytic enzymes by *Trichoderma* spp. contributes to their efficacy in suppressing root rot caused by *Phytophthora capsici* [18,33], suggesting that a similar mechanism acts against *P. ramorum*.

The dual-culture assays illuminated the complex relationship between *Trichoderma* growth rates and their biocontrol efficacy. While the commercial product SoilGard exhibited the highest rate of overgrowth and greatest lethal effect against *P. ramorum*, it was not statistically different from other isolates with varying growth rates. This underscores that while rapid growth aids in competition for space, it is not the sole determinant of biocontrol efficacy; other mechanisms like antibiosis are also crucial. Moreover, the observation that overgrowth did not always result in a lethal effect suggests that mechanisms beyond mycoparasitism, such as antibiosis, are involved in the biocontrol of *P. ramorum*.

The analysis of culture metabolites produced under starvation conditions provided further insights into the biocontrol mechanisms of *Trichoderma* isolates. *Trichoderma* spp. produce a variety of antibiotic-like substances and secondary metabolites that inhibit pathogen growth. These include compounds like Trichodermin and viridin [33] and volatile organic compounds (VOCs) with antimicrobial properties, contributing to antibiosis [10]. We found that several isolates, notably *T. polysporum*, produced antagonistic metabolites that significantly inhibited *P. ramorum* growth by more than 95%, emphasizing the role of antibiosis.

The results for *T. polysporum* were inconsistent between the two assays. While the microplate assay showed strong antibiosis (Figure 5), the dual-culture assay (Figure 4a,b) suggested limited mycoparasitic activity. This limited mycoparasitic activity may result from the slow linear growth rate of *T. polysporum* (Figure 3), suggesting that rapid growth is essential for effective mycoparasitism. Therefore, antibiosis through metabolite production was found to be a critical biocontrol mechanism of *Trichoderma* isolates against *P. ramorum*, especially for isolates with slower growth rates.

This study evaluated the biocontrol potential of various *Trichoderma* isolates against *P. ramorum* using several methodologies. Growth-rate studies at 20 °C served as a useful screening tool, generally correlating with indicators of biocontrol efficacy like overgrowth, lethal effect, and percentage inhibition. The dual-culture assays assessed direct antagonism by *Trichoderma* isolates through mycoparasitism. They provided insights into specific mechanisms of action, particularly the abilities to overgrow and to kill established cultures of *P. ramorum*. However, the dual-culture assays were complex and time-consuming, limiting their practicality for large-scale screening of mycoparasitic activity.

To efficiently assess the antibiosis mechanism, we developed a new microplate assay evaluating the inhibitory effects of *Trichoderma* culture filtrates on *P. ramorum* zoospore germination and growth. This assay evaluates antibiosis by testing the ability of sterile culture filtrates from *Trichoderma* to inhibit the pathogen. Using measurements of relative light absorbance to quantify *P. ramorum* zoospore germination and growth, this assay allowed us to efficiently screen multiple *Trichoderma* isolates for antibiosis. This efficient method facilitates easy replication, minimizes media usage, and provides rapid results, making it suitable for large-scale screening of biocontrol agents based on antibiosis.

The assays revealed different mechanisms of action. Selecting species and isolates for field studies requires considering the type of application. For instance, in a forest or nursery, a living *Trichoderma* isolate could be used as a foliar spray to prevent *P. ramorum* spread from leaf lesions, or as a soil treatment to minimize splash dispersal or root infection. The microplate assay we developed allows the selection of *Trichoderma* isolates with active metabolites against *P. ramorum* that could be used as a sterile product, eliminating persistence risk after application. These choices may be influenced by the specific *P. ramorum* hosts and the regulatory framework for biocontrol products. Future research should unravel these interactions and mechanisms to enhance the practical application of *Trichoderma* to manage *P. ramorum* infections [12,34]. Understanding the potential mechanisms of action of these isolates will enable us to further screen and tailor candidate agents to meet the specific requirements of diverse applications. Future studies should evaluate field and nursery applications to develop practical biocontrol strategies.

## 5. Conclusions

The predominantly positive correlations among variables suggest that a combined variable is useful for ranking isolates by biocontrol activity. This combined variable represented the isolates’ general vigour and biocontrol efficacy. Integrating these methods provided a comprehensive assessment of *Trichoderma* isolates’ biocontrol capabilities and mechanisms. The growth-rate study at 20 °C served as an efficient initial screening method. Although labour-intensive, the dual-culture assays provided detailed insights into direct antagonistic interactions. The new microplate assay enabled rapid, high-throughput evaluation of metabolite-mediated inhibition. Together, these approaches emphasize the importance of using diverse assays to select isolates with multiple mechanisms of action under varied conditions.

Our results partially support the hypothesis that *Trichoderma* isolates’ growth rates correlate with their biocontrol efficacy against *P. ramorum*. While rapid growth rates are advantageous and contribute to the competitive suppression of *P. ramorum*, our findings underscore that other factors, including antagonistic metabolites and possibly other mechanisms, play significant roles in the biocontrol process [28,35]. Our results align with those of previous studies demonstrating the importance of rapid colonization and metabolite production in effective biocontrol agents [10,36,37].

Advancements in developing and testing methods for applying promising *Trichoderma* isolates to plants or soil are continually progressing. These findings advance our understanding of biocontrol mechanisms against *P. ramorum* and have broader implications for sustainable disease management in forestry and agriculture [12,13,34,37].

## Figures and Tables

**Table 1:** *Trichoderma* isolates screened in this study, with tree host, geographic location of collection, and name of collector or source company.

Accession	Species ^1^	Host	Location	Collected by/Source
PFC 5001	* polysporum *	Black spruce	Comox, BC	T. Osono
PFC 5002	* polysporum *	Douglas fir	Comox, BC	T. Osono
PFC 5003	* polysporum *	Black spruce	Comox, BC	T. Osono
PFC 5004	* hamatum *	Black spruce	Comox, BC	T. Osono
PFC 5005	* hamatum *	Black spruce	Comox, BC	T. Osono
PFC 5006	* hamatum *	Douglas fir	Comox, BC	T. Osono
PFC 5007	* viride *	Black spruce	Comox, BC	T. Osono
PFC 5008	* viride *	Black spruce	Comox, BC	T. Osono
PFC 5009	* viride *	Black spruce	Comox, BC	T. Osono
PFC 5010	* viride *	Douglas fir	Berms, SK	T. Osono
PFC 5011	* viride *	Black spruce	Berms, SK	T. Osono
PFC 5012	* viride *	Jack pine	Berms, SK	T. Osono
PFC 5013	* koningii *	Douglas fir	Groundhog, ON	T. Osono
PFC 5014	* koningii *	Black spruce	Groundhog, ON	T. Osono
PFC 5015	* koningii *	Douglas fir	Groundhog, ON	T. Osono
PFC 5016	* polysporum *	Douglas fir	Groundhog, ON	T. Osono
PFC 5017	* polysporum *	Douglas fir	Groundhog, ON	T. Osono
PFC 5018	* polysporum *	Black spruce	Groundhog, ON	T. Osono
PFC 5019	* viride *	Black spruce	Groundhog, ON	T. Osono
PFC 5020	* viride *	Douglas fir	Groundhog, ON	T. Osono
PFC 5021	* viride *	Black spruce	Groundhog, ON	T. Osono
PFC 5022	* koningii *	Black spruce	CPRS, QC	T. Osono
PFC 5023	* koningii *	Black spruce	CPRS, QC	T. Osono
PFC 5024	* koningii *	Douglas fir	CPRS, QC	T. Osono
PFC 5025	* polysporum *	Douglas fir	CPRS, QC	T. Osono
PFC 5026	* polysporum *	Black spruce	CPRS, QC	T. Osono
PFC 5027	* viride *	Black spruce	CPRS, QC	T. Osono
PFC 5028	* viride *	Black spruce	CPRS, QC	T. Osono
PFC 5029	* viride *	Black spruce	CPRS, QC	T. Osono
PFC 5030	* koningii *	Douglas fir	Nashwaak, NB	T. Osono
PFC 5031	* koningii *	Balsam fir	Nashwaak, NB	T. Osono
PFC 5032	* polysporum *	Douglas fir	Nashwaak, NB	T. Osono
PFC 5033	* polysporum *	Douglas fir	Nashwaak, NB	T. Osono
PFC 5034	* polysporum *	Black spruce	Nashwaak, NB	T. Osono
PFC 5035	* viride *	Douglas fir	Nashwaak, NB	T. Osono
PFC 5036	* viride *	Black spruce	Nashwaak, NB	T. Osono
PFC 5037	* viride *	Douglas fir	Nashwaak, NB	T. Osono
PFC 5092	* hamatum *	Douglas fir	Oregon, USA	M. Elliott
PFC 5093	* pseudokoningii *	Douglas fir	Oregon, USA	M. Elliott
PFC 5094	* saturnisporum *	Douglas fir	Oregon, USA	M. Elliott
PFC 5095	* virens *	Douglas fir	Oregon, USA	M. Elliott
PFC 5096	* virens *	Douglas fir	Oregon, USA	M. Elliott
PFC 5097	* harzianum *	Douglas fir	Oregon, USA	M. Elliott
PFC 5098	* virens *	Douglas fir	Oregon, USA	M. Elliott
PFC 5099	* pseudokoningii *	Douglas fir	Oregon, USA	M. Elliott
PFC 5100	* hamatum *	Douglas fir	Oregon, USA	M. Elliott
PFC 5101	* koningii *	Douglas fir	Oregon, USA	M. Elliott
PFC 5102	* virens *	Douglas fir	Oregon, USA	M. Elliott
PlantHelper™	* atroviride *			AmPac Biotech
T382	* hamatum *			Sylvan Bioproducts
SoilGard™	* virens *			Certis Biologicals

^1^ All are members of the *Trichoderma* genus.

## Data Availability

The original data presented in the study are openly available in FigShare at https://doi.org/10.6084/m9.figshare.27961578.v1 (accessed on 20 January 2025).
